# Efavirenz 400 mg daily remains non-inferior to 600 mg: 96 week data from the double-blind, placebo-controlled ENCORE1 study

**DOI:** 10.7448/IAS.17.4.19523

**Published:** 2014-11-02

**Authors:** Dianne Carey

**Affiliations:** The Kirby Institute, University of New South Wales, Sydney, Australia

## Abstract

**Introduction:**

ENCORE1 compared the efficacy and safety of reduced versus standard dose efavirenz (EFV) with tenofovir/emtricitabine (TDF/FTC) as first-line HIV therapy. The primary analysis at 48 weeks showed 400 mg EFV was safe and virologically non-inferior to 600 mg. This analysis explores over 96 weeks the durability of efficacy and safety.

**Materials and Methods:**

A multinational, double-blind, placebo-controlled, non-inferiority trial in treatment-naïve HIV-positive adults randomized to TDF/FTC plus reduced (400 mg, EFV400) or standard dose (600 mg, EFV600) EFV. The difference between proportions of participants with plasma HIV RNA (VL) <200 log_10_ copies/mL by intention-to-treat (ITT missing=failure) was compared using a non-inferiority margin of −10%. Non-inferiority was also examined in per protocol (PP) and non-completer = failure (NC=F) populations. Adverse events (AEs) and serious adverse events (SAEs) were summarized by treatment arm.

**Results:**

The ITT population comprised 630 patients (EFV400 = 321; EFV600 = 309); 32% were female; 37%, 33% and 30% were African, Asian and Caucasian, respectively. A total of 585 (EFV400 = 299; EFV600 = 286) completed 96 weeks on randomized therapy. At 96 weeks, proportions with VL <200 copies/mL were EFV400 (90.0%) and EFV600 (90.6%) (difference −0.6; 95% CI −5.2 to 4.0; *p*=0.72) demonstrating continued non-inferiority. Non-inferior efficacy was also observed for VL thresholds of <50 and <400 copies/mL irrespective of baseline VL (<100,000 versus ≥100,000 copies/mL). There was no between-arm difference in time to loss of virological response (>200 copies/mL) (*p*=0.47) or mean change from baseline VL (*p*=0.74). Mean change from baseline in CD4 T-cell counts at week 96 remained significantly higher for EFV400 than EFV600 (difference 25 cells/µL; 95% CI 2–48; *p*=0.03). There was no difference in the frequency or severity of AEs (EFV400 = 89.4%, EFV600 = 89.3%; difference 0.09; 95% CI −4.73 to 4.90; *p*=0.97). The proportions ever reporting an AE definitely or probably EFV-related were EFV400 (37.7%) and EFV600 (47.9%) (difference −10.2%; 95% CI −17.9 to −2.51; *p*=0.01). SAEs did not differ in frequency (EFV400 = 7.5%, EFV600 = 10.4%; difference −2.9%; 95% CI −7.3 to 1.6; *p*=0.20).

**Conclusions:**

Non-inferiority of EFV 400 mg to EFV 600 mg when combined with TDF/FTC as initial HIV therapy was confirmed at week 96. Both doses demonstrated similar safety profiles. These results support the use of a lower EFV dose as part of routine HIV management.

